# Supplementary material for: Mechanochemical Approach to Obtaining a Multicomponent Fisetin Delivery System Improving Its Solubility and Biological Activity
Source: Int J Mol Sci. 2024 Mar 25;25(7):3648. doi: 10.3390/ijms25073648 (PMC11011862; doi:10.3390/ijms25073648)
Supplement: Supplementary file 1 [file ijms-25-03648-s001.zip › ijms-2888506-supplementary.pdf]

International Journal of *Molecular Sciences*

MDPI

Supplementary materials

Mechanistic Approach to Obtaining a Muliticomponent Fisetin Delivery System Improving Its Solubility and Biological Activity

Natalia Rosiak [1], Ewa Tykarska [2] and Judyta Cielecka-Piontek [1,*]

[1] Department of Pharmacognosy and Biomaterials, Faculty of Pharmacy, Poznan University of Medical Sciences, 3 Rokietnicka St., 60-806 Poznan, Poland; nrosiak@ump.edu.pl (N.R.); jpiontek@ump.edu.pl (J.C.-P.)

[2] Department of Chemical Technology of Drugs, Poznan University of Medical Sciences, 3 Rokietnicka St., 60-806 Poznan, Poland; etykarsk@ump.edu.pl (E.T.)

* Correspondence: jpiontek@ump.edu.pl (J.C.P.); Tel.: +48 61 641 83 95.

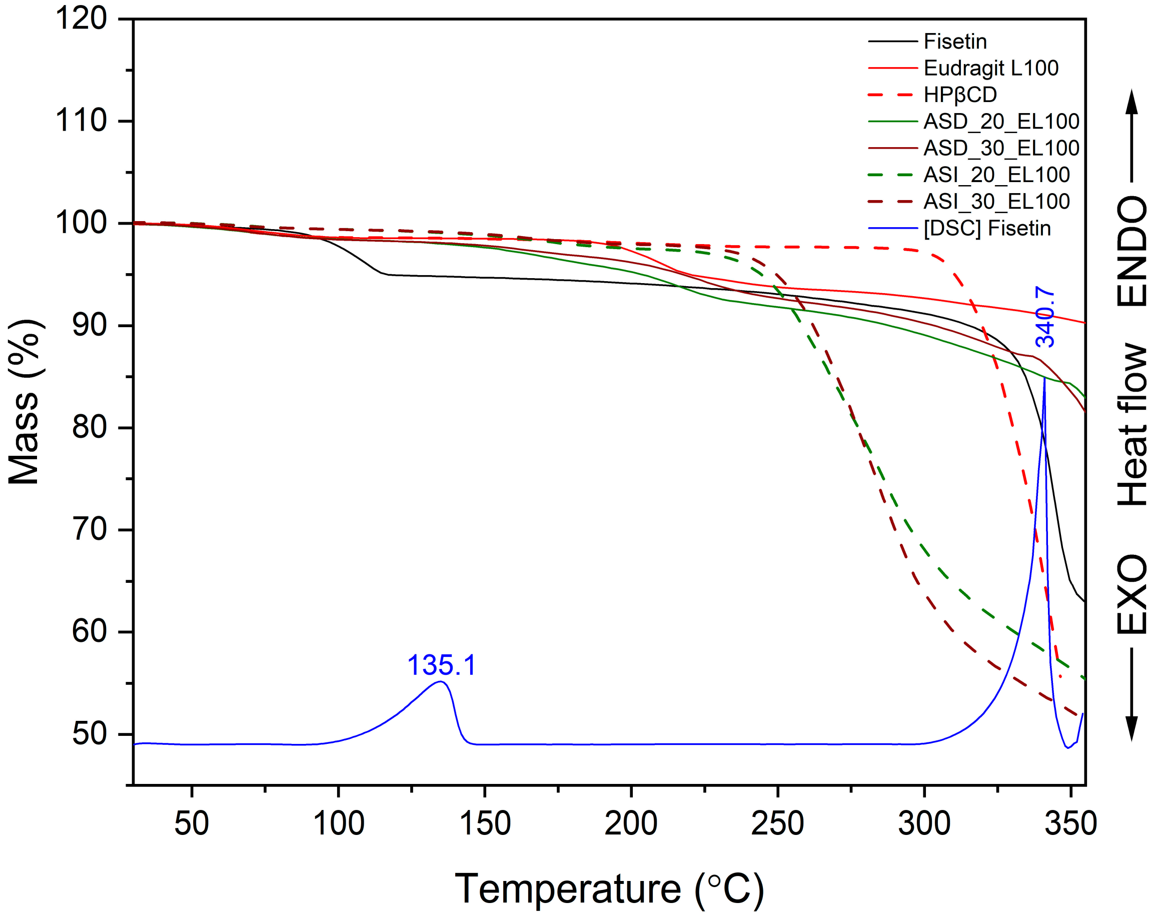

Figure S1. TG and DSC analysis: TG thermograms of neat compounds fisetin (FIS), Eudragit® L100 (EL100), 2-Hydroxypropyl-β-cyclodextrin (HPβCD); amorphous solid dispersion of FIS-EL100 (ASD), and amorphous solid inclusion of FIS-EL100-HPβCD (ASI); DSC thermogram recorded during the first heating scan for fisetin (blue line).

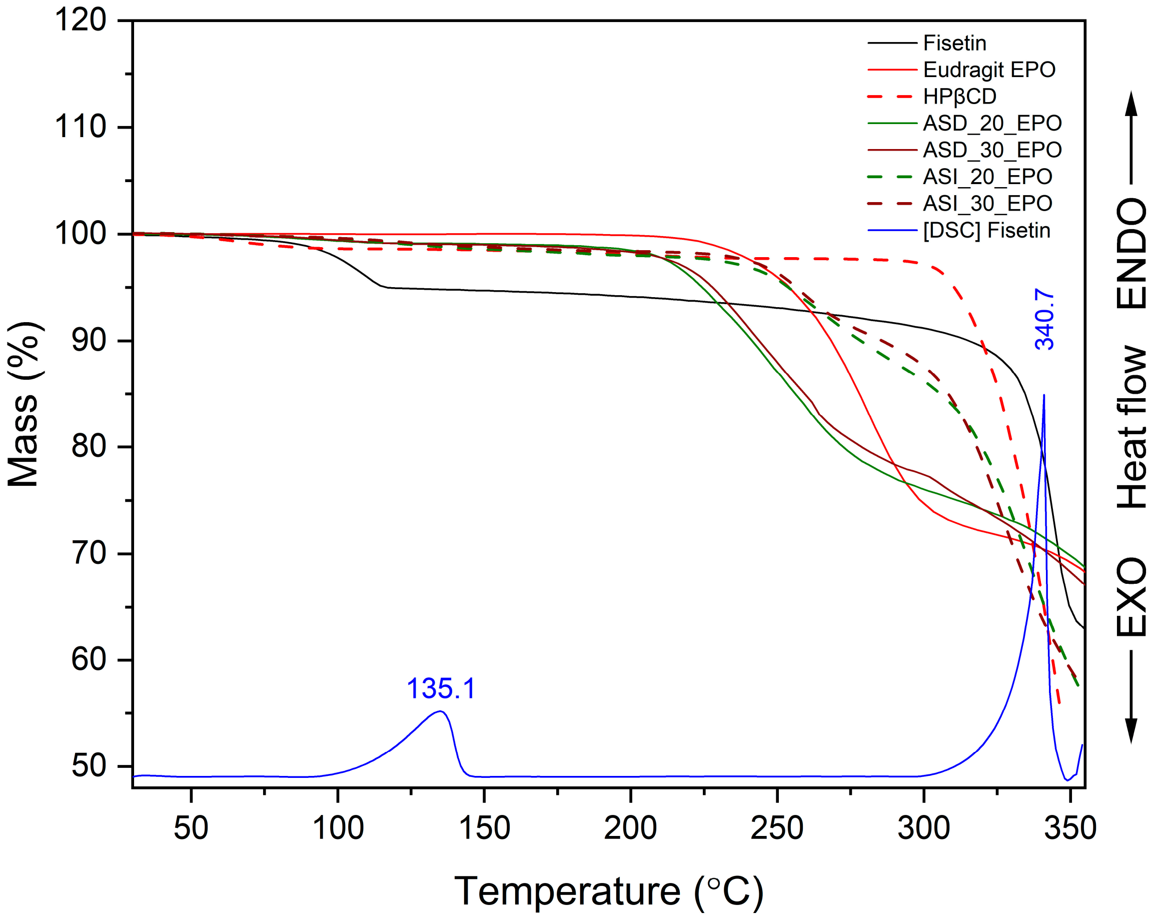

Figure S2. TG and DSC analysis: TG thermograms of neat compounds fisetin (FIS), Eudragit® L100 (EL100), 2-Hydroxypropyl-β-cyclodextrin (HPβCD), amorphous solid dispersion of FIS-EPO (ASD), and amorphous solid inclusion of FIS-EPO-HPβCD (ASI); DSC thermogram recorded during the first heating scan for fisetin (blue line).

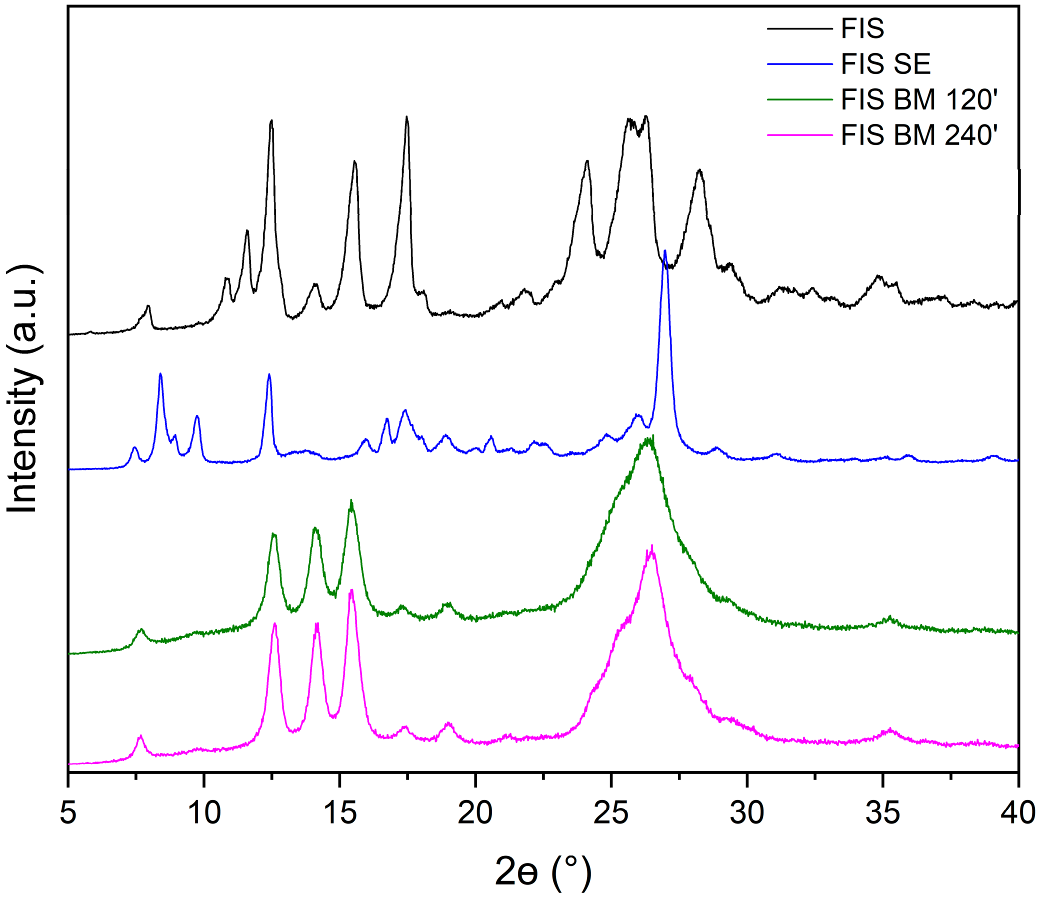

Figure S3. XRPD analysis: Diffractograms of FIS (neat fisetin), FIS SE (FIS after solvent evaporation), FIS BM 120′ and FIS BM 240′ (FIS after 120 and 240 minutes of milling, respectively).

Description of procedures:

FIS SE: 100 mg of FIS was added to a conical flask containing 25 mL of ethanol and placed in an ultrasound bath for about 2 min to obtain a lucid solution. The FIS ethanolic solution was poured into a round 50 mL bottom flask and placed in a rotary evaporator (Buchi, Switzerland) to remove ethanol under reduced pressure. The water bath was heated up to 50 °C. The process took enough time to dry the content of the flask visually. The sample was removed from the flask using a metal spatula.

FIS BM: 100 mg of FIS and two stainless steel balls with a diameter of 10 mm were placed in a 25 mL stainless steel jar. FIS milled at room temperature at 30 Hz for 120 minutes (FIS BM 120′) and 240 minutes (FIS BM 240′) on a Retsch MM-400 mixer mill machine (Mixer Mill, MM400, RETSCH, Bologna, Italy).

Table S1. Selected characteristic vibrational bands of fisetin (FIS) Eudragit® L100 (EL100), and amorphous solid dispersion of FIS-EL100 (ASD_EL100). Assignments bands were made based on literature [1–4]

Copyright: © 2024 by the authors. Licensee MDPI, Basel, Switzerland. This article is an open access article distributed under the terms and conditions of the Creative Commons Attribution (CC BY) license (https://creativecommons.org/licenses/by/4.0/).

FIS [cm⁻¹]	EL100	ASD_20_EL100 [cm⁻¹]	ASD_30_EL100 [cm⁻¹]	Assignments
627		621	621	OCCC t (A) oop + CCOC t (A) oop
675		673	672	CCO b (B, C)
770		775	773	CO s (B)
789		↓		HCCC t (B)
808		*	*	HCCC t (A)
854		847	847	HCCC b (A)
872		*	*	HCCC b (A)
935		↓		CCC b (A, B, C)
974		*	*	CCCH t (A)
1018		#	#	CCO b (C–B) + HCC b (B)
1117		*	*	HCC b (B) + COH b (B) + (4′–OH)
1132		*		COH b (A)
	1153	1155	1155	–C–O–C s
1163		*	*	CC s (C)
	1192	↑	↑	C–O vibration of carboxylic acid
1269		*	*	CC s (A, B) + CO s (A)
1329		1325 ↓	1325 ↓	CC s (B) + COH b (B) + (3′–OH; 4′–OH)
1437		*	*	COH b (B) + HCC b (B) + CCC b (B) + CCO b (A)
	1449	#	#	CH_3
1476		*	*	HCC b (A)
	1481	*	*	CH_x
1524		1508 ↓ #	1508 ↓ #	C–C s
1568		*	*	CC s (A, A–C) + CC s (C)

				(C2=C3) + C=O s (C)
1601		#	#	CC s (B)
1628		*	*	CC s (A) + C2=C3 s (C)
	1705	1699	1701	C–O s vibration of carboxylic ester
	1724	↓	↓	C=O s vibration groups of carboxylic acids
3246		*	*	CH stretching (A)
3346		*	*	CH stretching (B)
3518		*	*	OH group
3551		*	*	OH group

*Legend: # – shape change, * – band disappearance, ↑ – intensity increase, ↓ – intensity decrease, A, B, C – ring, b - bending, oop out of the plane, s - stretching, t - torsion.*

Table S2. Selected characteristic vibrational bands of fisetin (FIS) Eudragit® EPO (EPO), and amorphous solid dispersion of FIS-EPO (ASD_EPO). Assignments bands were made based on literature [1–6]

FIS [cm⁻¹]	EPO	ASD_20_EPO [cm⁻¹]	ASD_30_EPO [cm⁻¹]	Assignments
627		621	621	OCCC t (A) oop + CCOC t (A) oop
675		671 ↓	671 ↓	CCO b (B, C)
700		704 ↓	704 ↓	CCCC t (A, C-B) + CCOC t (A-C, C-B) oop + OCCC t (C, B) oop
770		773	773	CO s (B)
789		*	*	HCCC t (B)
808		*	*	HCCC t (A)
822		819 #	819 #	
854		847	847	HCCC b (A)
872		*	*	HCCC b (A)
935		*	↓	CCC b (A, B, C)
974		*	*	CCCH t (A)
1018		1015 ↓	1015 ↓	CCO b (C–B) + HCC b (B)
1117		1120 ↓	1120 ↓	HCC b (B) + COH b (B) + (4′–OH)
1132		*	*	COH b (A)
	1144	1146	1146	C–N s of aliphatic amine and/or C–O s of ester [7] or –C–O–C s
1163		*	*	CC s (C)
1206		*	*	CO s (A, C) + COH b (A) + (7-OH)
	1240	↓	↓	C–O s of ester
1269		*	*	CC s (A, B) + CO s (A)
	1269	1267 ↑	1267 ↑	C–O s of ester
1283		*	*	HCC b (B)
1329		↓	↓	CC s (B) + COH b (B) + (3′–OH; 4′–OH)
1437		*	*	COH b (B) + HCC b (B) + CCC b (B) + CCO b (A)
	1454	↑ #	↑ #	C–H b of methyl
1476		*	*	HCC b (A)
1524		*	*	C–C s
1568		*	*	CC s (A, A–C) + CC s (C) (C2=C3) + C=O s (C)
1601		1607	1605	CC s (B)
1628		*	*	CC s (A) + C2=C3 s (C)
	2770	↓	↓	dimethyl amino groups
	2822	↓	↓	alkene C–H stretching
	2949	2953	2953	hydrocarbon chain
3246		*	*	CH stretching (A)
3346		*	*	CH stretching (B)
3518		*	*	OH group
3551		*	*	OH group

*Legend: # – shape change, * – band disappearance, ↑ – intensity increase, ↓ – intensity decrease, A, B, C – ring, b - bending, oop out of the plane, s - stretching, t - torsion.*

References

1. Sip, S.; Rosiak, N.; Sip, A.; Żarowski, M.; Hojan, K.; Cielecka-Piontek, J. A Fisetin Delivery System for Neuroprotection: A Co-Amorphous Dispersion Prepared in Supercritical Carbon Dioxide. Antioxidants 2023, 13, 24, doi:10.3390/antiox13010024.
2. Rosiak, N.; Tykarska, E.; Cielecka-Piontek, J. The Study of Amorphous Kaempferol Dispersions Involving FT-IR Spectroscopy. Int. J. Mol. Sci. 2023, 24, 17155, doi:10.3390/ijms242417155.

3. Marković, J.M.D.; Marković, Z.S.; Milenković, D.; Jeremić, S. Application of comparative vibrational spectroscopic and mechanistic studies in analysis of fisetin structure. Spectrochim. Acta Part A Mol. Biomol. Spectrosc. 2011, 83, 120–129.
4. Awadeen, R.H.; Boughdady, M.F.; Zaghloul, R.A.; Elsaed, W.M.; Abu Hashim, I.I.; Meshali, M.M. Formulation of lipid polymer hybrid nanoparticles of the phytochemical Fisetin and its in vivo assessment against severe acute pancreatitis. Sci. Rep. 2023, 13, 19110.
5. Inam, S.; Irfan, M.; Lali, N.U.A.; Khalid Syed, H.; Asghar, S.; Khan, I.U.; Khan, S.-U.-D.; Iqbal, M.S.; Zaheer, I.; Khames, A.; et al. Development and Characterization of Eudragit® EPO-Based Solid Dispersion of Rosuvastatin Calcium to Foresee the Impact on Solubility, Dissolution and Antihyperlipidemic Activity. Pharmaceuticals 2022, 15, 492, doi:10.3390/ph15040492.
6. Linares, V.; Yarce, C.J.; Echeverri, J.D.; Galeano, E.; Salamanca, C.H. Relationship between degree of polymeric ionisation and hydrolytic degradation of Eudragit® E polymers under extreme acid conditions. Polymers (Basel). 2019, 11, 1010.
7. Lin, S.-Y.; Cheng, W.-T.; Wei, Y.-S.; Lin, H.-L. DSC-FTIR microspectroscopy used to investigate the heat-induced intramolecular cyclic anhydride formation between Eudragit E and PVA copolymer. Polym. J. 2011, 43, 577–580, doi:10.1038/pj.2011.15.

Disclaimer/Publisher's Note: The statements, opinions and data contained in all publications are solely those of the individual author(s) and contributor(s) and not of MDPI and/or the editor(s). MDPI and/or the editor(s) disclaim responsibility for any injury to people or property resulting from any ideas, methods, instructions or products referred to in the content.
